# Deterioration, Compensation and Motor Control Processes in Healthy Aging, Mild Cognitive Impairment and Alzheimer’s Disease

**DOI:** 10.3390/geriatrics6010033

**Published:** 2021-03-23

**Authors:** Gabriel Poirier, Alice Ohayon, Adrien Juranville, France Mourey, Jeremie Gaveau

**Affiliations:** 1 INSERM U1093-CAPS, Université Bourgogne Franche-Comté, UFR des Sciences du Sport, F-21000 Dijon, France; aliceohayon21@gmail.com (A.O.); ajuranvefom@gmail.com (A.J.); France.Mourey@u-bourgogne.fr (F.M.); jeremie.gaveau@u-bourgogne.fr (J.G.); 2 Espace d’Étude du Mouvement—Étienne Jules MAREY, Université Bourgogne Franche-Comté, UFR des Sciences du Sport, F-21000 Dijon, France

**Keywords:** aging, motor control, compensation, deterioration, Alzheimer’s, cognition

## Abstract

Aging is associated with modifications of several brain structures and functions. These modifications then manifest as modified behaviors. It has been proposed that some brain function modifications may compensate for some other deteriorated ones, thus maintaining behavioral performance. Through the concept of compensation versus deterioration, this article reviews the literature on motor function in healthy and pathological aging. We first highlight mechanistic studies that used paradigms, allowing us to identify precise compensation mechanisms in healthy aging. Subsequently, we review studies investigating motor function in two often-associated neurological conditions, i.e., mild cognitive impairment and Alzheimer’s disease. We point out the need to expand the knowledge gained from descriptive studies with studies targeting specific motor control processes. Teasing apart deteriorated versus compensating processes represents precious knowledge that could significantly improve the prevention and rehabilitation of age-related loss of mobility.

## 1. Introduction

The proportion of old adults in the world population is growing rapidly [1]. This phenomenon results in an increased prevalence of age-onset neurological conditions, thereby implying a heavy socio-economic burden [2,3,4,5]. An important part of this burden is due to mobility impairments [6,7,8], which have significant repercussions on functional autonomy and predict deleterious health outcomes [9,10,11,12,13,14]. Effectively improving the prevention and rehabilitation of mobility loss requires a good understanding of motor function in healthy and pathological older populations. This article presents a concise review of the literature on motor function in healthy aging and two often-associated neurological conditions, i.e., mild cognitive impairment (MCI) and Alzheimer’s disease (AD). More specifically, this review aims at promoting studies that offer a mechanistic understanding of motor function as an expansion of purely descriptive studies.

## 2. Deterioration and Compensation during Healthy Aging

Studies investigating age-related modifications of motor function have first focused on peripheral neuromuscular factors—see [15] for a review. It is now well known that alteration of the central nervous system (CNS) also significantly contributes to motor dysfunction in older adults [16,17,18,19]. Modifications of the CNS can be structural and/or functional [17,20]. Structural modifications refer to the degradation of brain structures with aging (e.g., cortical atrophy), while functional modifications denote changes in how these structures operate in the act of motor control. Some functional changes occurring with aging may not systematically be detrimental to motor performance. Some changes may correspond to a function degradation, while others may benefit motor performance and represent compensations for function degradations [17,20].

Cabeza et al., (HAROLD model; [21,22]) first raised the concept of functional compensation in older adults. Using positron emission tomography and functional magnetic resonance imagery, these authors investigated brain activations in young and older adults during various cognitive tasks. According to their performances, Cabeza et al. (2002) separated older participants into high- and low-performing groups. Their results showed that whereas pre-frontal cortex recruitment was right-lateralized in young participants and low-performing older adults, it was bilateral in high-performing older adults. The authors proposed that this additional recruitment in high-performing older adults compensates for age-related brain degeneration. Other studies, however, have proposed that such increased brain activity may reflect an inability to select appropriate brain regions [23,24].

**Figure 1:**
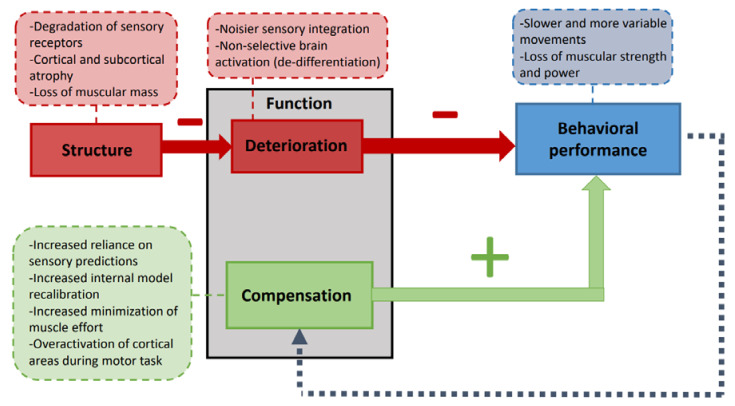
A model of deterioration and compensation of the motor function with aging. Adapted from Papegaaij et al. (2014). Aging alters the structure of the sensorimotor system, causing functional deteriorations (i.e., deterioration in how these structures act during motor tasks) and decreased behavioral motor performances. Decreased performance triggers the need for functional compensations (modifications in how brain structures act to attain motor goals) to maintain behavioral performance. Dashed boxes provide examples of deteriorated and compensating modifications. Examples of age-related structure alterations: degradation of sensory receptors [48,49,50], cortical atrophy [51,52,53], and loss of muscular mass [54]. Examples of functional deterioration: noisier sensory integration [48,50] and nonselective brain activation [17,23]. Examples of behavioral modifications: slower and more variable movements [46,47] and loss of muscular strength and power [45,54]. Examples of functional compensations: increased reliance on sensory predictions [55,56,57], increased internal model recalibration [58], increased minimization of muscle effort [59], and overactivation of cortical areas during motor tasks [26].

Increased brain activation in older individuals is also observed during motor tasks. Increased activation of contralateral motor areas (premotor cortex, primary motor cortex, and supplementary motor area) has been consistently reported [25,26,27,28,29,30,31,32]. Older individuals may also recruit additional cortical and subcortical areas, including the ipsilateral motor cortex, during motor preparation and execution [26,27,28,29,31,32,33,34,35,36,37,38]. Some studies suggest that such increased brain activations are nonselective and do not impact behavioral performance (dedifferentiation hypothesis; [17,22,23,33,38,39,40,41]). Others, observing significant correlations between brain activations and behavioral performance, support the view that increased activations compensate for age-related deterioration [26,27,28,37,42,43,44]. Figure 1 displays a graphical model (adapted from Papegaaij et al. (2014)) of deterioration and compensation mechanisms in aging. 

Overall, in the past two decades, the concept of functional motor compensation in older adults has been a controversial subject of several brain imagery and stimulation studies that have investigated brain correlates of motor performance. These performances decline with age due to peripheral neuromuscular as well as central factors [15,17] and include, amongst others, decreased muscle force [45], decreased movement speed [46], and increased variability [47]. 

## 3. Mechanistic Studies of Motor Function in Healthy Aging

Insofar as modified behavioral performances result from the combination of deteriorated and compensating motor processes in older adults, coarse performance metrics—e.g., force, speed, or precision—are limited in their ability to study compensation mechanisms. They can only measure the net result of compensation and deterioration. It is only recently that behavioral studies have started to consistently and specifically address the concept of functional compensation in older adults. Using paradigms that allowed one to target and isolate specific motor control processes, several studies have highlighted possible compensation mechanisms. Below, we list a few examples. 

Wolpe et al. (2016) [55] investigated sensory attenuation—the reduction in the perceived intensity of self-generated actions—via a force-matching task in a population-based cohort (*n* = 325; 18–88 years). Results showed increased sensory attenuation in older adults. Furthermore, inter-participant analyses revealed that the size of this effect was proportional to the participants’ sensitivity. This result was also associated with measures of the structural and functional connectivity of the pre-supplementary motor area. As increased sensory attenuation suggests a stronger weighting of predictive signals, these results support increased reliance on predictive signals to compensate for noisier sensory signals. 

Helsen et al. (2016) [56] investigated the accuracy of wrist-aiming movements with and without visual feedback, along with proprioceptive acuity in young and older adults. Results showed similar accuracy across age groups for visual and non-visual conditions, but older adults exhibited longer movement times and made more corrective sub-movements. Although proprioceptive acuity decreased in older adults, it did not predict aiming behavior. These results also argue for a strategy where older adults increase their reliance on predictive control to compensate for decreased proprioceptive acuity. 

Hoellinger et al. (2017) [57] asked young and older adults to make ecological reach-grasp-lift movements with an object whose weight randomly varied across trials. The authors then performed detailed kinematic analysis. Among other results, older adults exhibited shorter movement times and spent more time accelerating than young adults. Theoretical simulations explained these results as a strategy where older adults overestimate the object’s weight and preferentially rely on predictive processes to compensate for diminished sensory acuity. 

Poirier et al. (2020) [59] investigated vertical arm movements in young and older adults. The authors used a paradigm allowing them to test how motor planning adapts motor patterns to the gravitational environment. Young adults are known to use an optimal strategy that minimizes muscle effort. Directional asymmetries can quantify this strategy, i.e., differences between upward and downward movements [60,61,62,63]. Poirier et al. (2020) observed that young and older participants exhibited qualitatively similar directional asymmetries, thereby indicating that older adults can unfold the same effort-related optimization strategy as young adults. Still, the size of the directional asymmetry was more extensive in older participants than in young ones, further suggesting that subtle modifications of effort minimization processes may exist with age. Grounded on previous modeling work about gravity-related optimal motor planning [60,61,62,63], increased directional asymmetries in older adults support increased optimization of gravity effects—i.e., increased minimization of muscle effort—to compensate for muscle force decrease. During postural tasks, however, results from other studies may instead support a strategy where older adults emphasize equilibrium maintenance rather than effort minimization [64].

Previous studies have shown that aging causes a reduction in motor adaptation [65,66,67,68,69,70]. It was, however, uncertain whether this decreased motor adaptation was due to explicit (i.e., cognitive) or implicit (i.e., internal model recalibration) components of motor adaptation. Vandevoorde and Orban de Xivry (2019) [58] using paradigms assessing both components and found that reduced motor adaptation is due to a decreased cognitive component in older adults. Internal model recalibration was intact or even increased in older adults. The authors, therefore, proposed that increased internal model recalibration could compensate for the decreased cognitive component. 

Such studies offer a deeper understanding of motor control modifications in the aging population. Targeting precise motor control processes demonstrates that some age-related modifications represent compensations for other deteriorated processes. For example, contrary to the ancient view, these studies show that predictive processes are still functional in older adults. In addition, predictive processes seem to be favored over feedback processes to compensate for unreliable sensory signals. Teasing apart deteriorated versus compensating processes represents invaluable knowledge that could significantly improve the prevention and rehabilitation of age-related loss of mobility [9,71]. Such knowledge is also crucial to effectively designing and interpreting neurophysiological investigations [72,73]. 

## 4. Motor Function Studies in Pathological Aging: Alzheimer’s Disease (AD) and Mild Cognitive Impairment (MCI) 

AD is an age-related neurodegenerative disease associated with neurofibrillary tangles and amyloid plaques, causing loss of memory, general cognitive decline, and eventually dementia. The onset of AD may be preceded by MCI, known as a transitional stage between healthy aging and AD and characterized by soft cognitive symptoms that do not functionally impact daily life [74]. These pathologies have long been considered as pathologies that mostly affect cognitive functions. It is now notoriously known that mobility capacities also are worse in AD and MCI populations than age-matched cognitively unimpaired older adults. Overall, studies have reported slower gait speed, as well as a shorter and more variable stride [75,76,77,78,79,80,81,82,83,84,85,86,87]. Balance and postural control are also impaired [77,78,88,89,90,91,92,93,94,95], and kinematic studies investigating handwriting and finger-tapping movements have reported fine motor control deterioration in MCI and AD patients [96,97,98,99,100].

The cognitive neuroscience concept of reserve, maintenance, and compensation [101] and cognition–action theories [17] can propose a possible explanation for the motor decline in AD and MCI. Reserve is the accumulation of brain resources during the life span, maintenance is the preservation of these resources via constant recovery, and compensation is the deployment of new resources to execute a given constant task demand. The inability to compensate for age-related degeneration could be framed as a lack of reserves or their deterioration. Some studies suggest that higher cognitive reserves delay the onset of cognitive symptoms in AD [102,103]. These reserves may compensate for brain degeneration at the beginning of the pathology, but with the progression of deterioration, reserves become insufficient to compensate for the pathology’s effects. Thus, compensation is no longer possible, and symptoms appear. A parallel can be made for motor control deteriorations in AD and MCI. As brain reserves decline, age-related changes can no longer be compensated and become more salient (see Figure 1). Evidence showing that cognitive and motor regions are functionally interdependent supports this view [104]. During challenging motor tasks, older adults also recruit additional brain regions that were viewed as mainly cognitive regions [28,105]. The strengthening of reserves therefore seems essential in the prevention of pathological aging. The simultaneous execution of a motor and a cognitive task is an excellent example of what can be tested during aging to probe the evolution of cognition–action interactions and inform geriatric rehabilitation [106].

Studies have shown that modifications of sensorimotor functions may precede the onset of dementia [91,107,108] and that the presence of motor dysfunction could predict adverse outcomes in AD patients, such as fall risk [9,10,11,12,13,14,84,85,108,109,110,111]. In MCI, studies have also reported that motor function impairments are predictive of a higher risk of developing AD [79,98,108,112]. Aggarwal et al. (2006) investigated lower limb motor function in an aged longitudinal cohort study. They quantified lower limb performance with an index, including walking speed, sit-to-stand speed, and balance time on unipodal and bipodal tests. Lower performance was associated with an increased risk of developing AD. 

Because motor deficits, or at least their detection, seem to precede cognitive impairment, one could use them as predictive diagnostic tools [84,91,98,113]. For example, in a recent study, Ehsani et al. (2020) asked MCI patients, AD patients, and healthy controls to execute repetitive elbow flexion on a dual task (counting backward by ones and threes). Logistic ordinal models were able to predict the different groups’ cognitive status from measures of elbow angle variability and angular velocity. These models had good sensitivity and specificity and, thus, could be used to detect cognitive impairment in older adults. Such tools could also predict cognitive capacities’ evolution [98,108,114,115,116]. For example, Chou et al., 2019 [117] evaluated the handgrip strength and gait speed of participants aged 60 and over. Over a 10-year period, they correlated the results with those of cognitive tests (the Mini-Mental Score Examination and the Digit Symbol Substitution Test). Motor measures were effective at predicting cognitive decline. All these results demonstrate motor control impairments in AD and MCI patients. They also show that studying these impairments is of particular interest for diagnosis purposes. These studies, however, mostly assessed global motor function rather than specific motor control processes. Thus, they can mostly observe the net result of degraded and compensating processes.

## 5. Final Remarks and Futures Perspectives

As illustrated above with regard to healthy aging, studying motor control processes could allow a precious understanding of compensation versus deterioration mechanisms. In addition, in pathological aging, this could (i) improve the efficacy of sensorimotor measures for diagnosis purposes, (ii) inform prevention programs about the loss of mobility, (iii) benefit personalized medicine for frail older adults who notoriously present with heterogeneous deficiencies [118], and (iv) generate new hypotheses about the physiopathology/etiology of neurological diseases. There is a growing literature probing specific motor control processes in healthy aging but a critical lack of such studies on pathological aging. Building future studies within the deterioration versus compensation framework may strongly benefit aging research and care. It is urgent to promote such studies [72]. 

## Data Availability

Not applicable.
